# Assessment of dialysis adequacy: beyond urea kinetic measurements

**DOI:** 10.1007/s00467-018-3914-6

**Published:** 2018-03-26

**Authors:** Lesley Rees

**Affiliations:** Renal Office, Gt Ormond St Hospital for Children NHS Foundation Trust, WC1N 3JH, London, UK

**Keywords:** Dialysis, Adequacy, Optimum dialysis

## Abstract

Adequacy of dialysis is a term that has been used for many years based on measurement of small solute clearance using urea and creatinine. This has been shown in some but not all studies in adults to correlate with survival. However, small solute clearance is just one minor part of the effectiveness of dialysis and in fact ‘optimum’ dialysis, rather than ‘adequate’ dialysis is what most paediatric nephrologists would want for their patients. Additional ways to assess the success of dialysis in children would include dialysis access complications and longevity, preservation of residual kidney function, body composition, biochemical and haematological control, nutrition and growth, discomfort during the dialysis process and psychosocial adjustment including hospitalisation and school attendance. These criteria need to be balanced against a dialysis programme that has the least possible adverse effects on quality of life.

## Introduction

The concept of dialysis adequacy was developed in the 1980s after it was shown that the blood urea level influenced survival in adult patients and so the concept of urea clearance and Kt/V_urea_ was born. Classically measures of dialysis adequacy since have been based on small solute clearance using urea and creatinine and are defined as the minimum urea clearance and nutritional intake that prevent adverse outcomes [1].

There are innate problems with the interpretation of these formulae, especially in children. The optimum Kt/V for children is not known, but it is generally accepted that the delivered dose aimed for in adults should be a minimum. Normalisation for body size using V (urea distribution volume, equating to total body water) means that smaller patients will need a relatively lower dialysis dose to achieve the same Kt/V target. However, the metabolic activity and protein intake might even be greater in these smaller patients, so it has been suggested that normalisation of Kt to surface area (SA) would be preferable. In prepubertal children, the ratio of V to SA is lower than in adolescents so that if a SA-based denominator were to be adopted for dialysis dosing, most children below 10 years of age would receive significantly less dialysis than adolescents, and would require haemodialysis sessions lasting 6 to 8 h or for the youngest children, treatments given more frequently than three times per week [2].

As well as SA, resting energy expenditure (REE) and total energy expenditure (TEE) have been suggested as alternative parameters to replace V. Uraemic toxins come from metabolic activity within body cells as well as from the gut, and are increased by physical activity, suggesting that adjustments for TEE alongside incorporation of a factor for protein intake, could be used instead of V [3, 4].

However, small solute clearance (represented by urea) is just one part of the effectiveness of dialysis and does not measure clearance of larger and possibly more important middle molecules that move more slowly, or protein-bound molecules that may be very difficult to remove. Although it remains an important measure, particularly as it has recently been suggested that urea is not the inert molecule it was thought to be, and has toxic effects in its own right [5], there is much more to dialysis than simply urea clearance. Something is adequate if it is sufficient or acceptable, but is that what we want for our patients? A preferable way forward would surely be to provide the best possible dialysis regimen. But how do we decide if the dialysis we are providing is either adequate or optimum?

## Benefits of intensive dialysis versus quality of life

Clearly, the purpose of dialysis is to extend life and indeed without it death would ultimately occur. However, quality of life is generally not improved in the setting of dialysis. Quality of life should be, but often is not, a major influence on the prescribed dialysis-related care and responsibilities. With good dialysis, the child will feel better with less uraemic symptoms, and there are obvious implications for long-term morbidity and survival. However, this can cause a conflict as escalating dialysis programmes may impact on quality of life. Adult nephrologists are moving towards incremental dialysis, building up hours of treatment as residual kidney function (RKF) falls. This requires careful monitoring of urine output combined with the use of urea kinetic equations to calculate the time on dialysis required to obtain ‘adequacy’ [6]. This may not be appropriate in children, in whom the values for neither ‘adequate’ nor ‘optimum’ urea clearances are known, and whose metabolic needs are likely to mean higher clearance requirements.

If we take, for example, a 10-year old on three times per week in centre HD. If it takes them an hour to get to the centre, 15 min to be put onto the machine, 4 h on the machine, 15 min to come off again and another hour to get home; and if we assume they are awake for 12 h a day, this represents over half of their waking day and, if dialysed 3 days out of seven, over 20% of their week. We know that many children live a long way from their HD centre so this may be an underestimate. Some children do not feel well straight after their session and for some hours thereafter. This concept of ‘treatment time’ has been used in adult patients to recommend that the increased life expectancy offered by more frequent dialysis is outweighed by the impact on their quality of life, and to the development of the concept of an ‘effective survival time’ which is a calculation of life expectancy minus the time required for treatment and post treatment recovery [7]. Although treatment time may be important in an adult with a relatively short life expectancy, and in whom depression has been independently linked with survival [1], it is not a justified approach in children with a lifetime of renal replacement therapy (RRT) ahead of them. Of course these arguments do not apply to any patient on home dialysis therapies.

## When should dialysis be started?

Our first decision is, when is the optimum time to start dialysis in our patients? Will they have a better quality of life managed with strict dietary control and medications without dialysis, or will they feel and thrive better on dialysis? In some situations, the timing of the decision is clear: the patient with declining urine output, salt and water retention causing difficult to treat hypertension, or uraemic symptoms, abnormal biochemistry and poor growth. However, much can be done with good dietary control and medications, particularly in patients with Congenital Anomalies of the Kidney and Urinary Tract (CAKUT), who often maintain high urine outputs despite a very low glomerular filtration rate (GFR) so that blood pressure (BP) and potassium are usually normal. A useful aid in predicting the likely timing of the onset of ESKD is the Kidney Failure Risk Equation, which uses the age, sex, estimated glomerular filtration rate (eGFR), urine albumin/creatinine, calcium, phosphate, bicarbonate and albumin to predict the risk of progression to ESKD over 5 years [8]. There is currently very little evidence in children to tell us whether early initiation of dialysis will or will not improve quality of life and outcome, although in adults a randomised, controlled trial has found no benefit in starting dialysis before a GFR of 10 ml/min/1.73m^2^ [9]. A recent study from Turkey comparing early start of dialysis, defined as an eGFR of > 10 ml/min/1.73 m^2^, and late start, using an eGFR of < 7 ml/min/1.73m^2^, found no difference over approximately 3 years in left ventricular hypertrophy, inflammatory state or hospitalisation [10].

The difficulty in making this decision is evidenced by the wide variability of GFR at the start of dialysis around the world. Although the median GFR in the USA, Canada and Europe is 8 to 9 ml/min/1.73m^2^, the ranges are from below 5 to over 10 ml/min/1.73m^2^ [11–13]. In Canada, there has been a trend over the last two decades to starting dialysis at higher GFRs, with one third of the children initiating dialysis with an eGFR ≥ 10.5 ml/min/1.73 m^2^. The differences seem to be centre specific without any other clearly identifiable factor [13]. The majority of paediatric nephrologists believe that transplantation is the best form of RRT and would try to avoid dialysis altogether, so that waiting on the transplant list may contribute to the lower GFRs at start of dialysis seen in some patients.

## What would be the ideal mode of dialysis for our patients?

When the decision to start dialysis has been made, what is the best dialysis option for our patients that will improve both well-being and survival? The child is likely to have a different concept of optimum dialysis from their doctor. Children want a dialysis process that takes as little time as possible, is not painful and does not make them feel unwell, enables them to be at home with their family as much as they can and to be able to go out with their friends and get to school. They would like to have a free diet with no fluid restriction and no medications. Parents know this and when they are made aware of the benefits of home therapies are often prepared to take upon themselves the burden of home care and enforcement of all the rigours that go with it. The ideal dialysis that doctors want for their patients is different again: they want a child with dialysis access that is well functioning and long lasting without complications; with good native urine output that does not decline with time and treatment; who is maintained at their target weight and BP without antihypertensive therapy or left ventricular hypertrophy (LVH); who has no discomfort during the dialysis process and in particular no intradialytic hypotension; who is not anaemic or acidotic and in whom there is good control of potassium, calcium and phosphate and parathyroid hormone (PTH); who has good appetite, nutrition and growth with a satisfactory urea and albumin level and who feels well and attends school with good educational attainment and does not require hospital admissions. This must all be achieved with a dialysis programme that interferes as little as possible with day to day socialisation and schooling, and as little as possible with quality of life.

## Types of dialysis

Is there any evidence that any mode of dialysis is best, pointing us in the right direction for optimising treatments? In terms of survival, there is no difference at follow-up between in centre haemodialysis (HD) and peritoneal dialysis (PD), being 90% after 5 years for both modalities [14]

For those children lucky enough to have a family that is able to undertake the commitment required for a home PD or HD programme, the outlook for social integration is infinitely more positive. Home HD has not only social benefits but also improves patient well-being, nutrition, growth and biochemical control with commonly no need for a fluid restriction and minimisation of medications [15]. This, along with the opportunity for a full social and educational life, is likely to fit in best with the child’s concepts of optimum dialysis.

However, home dialysis places a huge burden and responsibility on the carers. The relentless daily tasks of preparing the machine, assessment of the fluid status of the child before the procedure and the procedure itself, can lead to carer stress and burnout. Interruption to sleep is common with overnight PD, which may need to be combined with overnight feeds. Children who need in centre HD are often those without such good home support, and may be more likely to require prolonged dialysis due to previous sensitisation from failed transplants. For them, early results from a comparative study of HD/haemodiafiltration (HDF) suggest benefits of HDF over HD on progression of CVD (personal communication from Dr. R Shroff). This work in children, along with studies in adults [16], should encourage us to consider HDF in our in centre patients.

## What ways do we have to improve the dialysis process and how can we assess whether optimum dialysis has been achieved? (Table 1)**.**

### Dialysis access

**Fig. 1:**
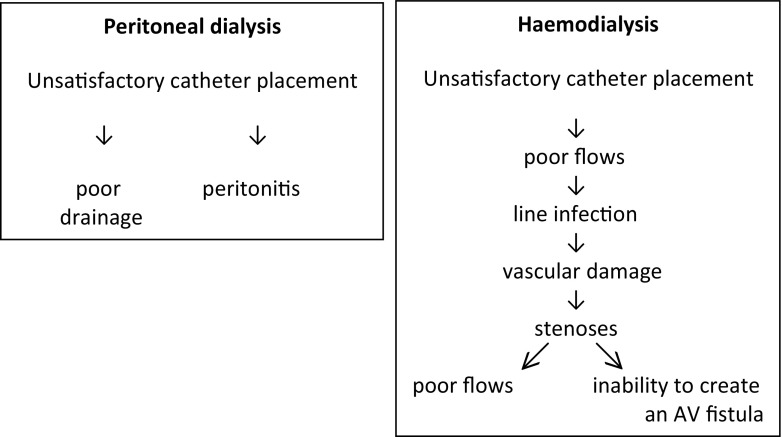
Ensuring optimal dialysis: access is crucial

For optimum dialysis, the first priority is access: without good access, dialysis will never even be adequate. Too often PD catheter placement is left to inexperienced staff when this is a skilled procedure which has implications not just for the short-term but for also long-term survival. Unsatisfactory catheter placement can lead to exit site and tunnel infection, leakage, poor drainage and peritonitis (Fig. 1). For HD, the priority for the policy of ‘fistula first’ is infrequently followed, and access chosen is all too often a central venous line (CVL). If not well placed, poor flows result in the need for frequent disconnections, flushing and fluid boluses with the consequent risks of introduction of infection and persistent fluid overload. Infection is followed by vessel stenosis which increases further the poor flows and reduces the chances of subsequent fistula formation (Fig. 1). Arteriovenous fistulae are associated with less infection, longer access survival and less hospitalisation. One important problem that has a detrimental effect on the fistula first policy is the lack of experienced surgical staff, particularly for the very small child. With good play therapy and the use of local anaesthetic creams, fistulae can be successfully used even in very young children [17]. The development of a dedicated clinic with a multidisciplinary team of specialised ultrasonographers, a nephrologist, a senior haemodialysis nurse and a vascular surgeon has encouraged the use of arteriovenous (AV) fistulae and their maintenance after creation [18]. It has to be remembered that every patient has only five lifelines: two arms, two legs and the peritoneum. If these are expended, then without access to transplantation, the future is bleak.

**Table 1:** What ways do we have to improve the dialysis process and how can we assess whether optimum dialysis has been achieved?

Optimum dialysis	How can this be assessed?
Well-functioning, long-lasting access with no complications	Compliance with ‘fistula first’ policy Access infection and access failure
Maintaining RKF	Urine output and use of diuretics
Target weight maintained, with normal BP without antihypertensives and no LVH	BP SDS, ECHO and number of antihypertensive medications
No discomfort during dialysis or intradialytic hypotension	Percentage with pain interfering with PD. Intradialytic weight gains and UF rates < 13 ml/kg/h
No anaemia, acidosis, or potassium, calcium, phosphate or PTH disturbance	Audit of haematological and biochemical control
Good nutrition and growth	Urea, albumin, Ht SDS, Wt SDS, head circumference SDS and pubertal development
No hospitalisations for complications	Hospitalisation rates
Psychosocial care provided and educational input	Access to social workers, psychologists and play therapists. Assessment of HRQoL-targeted educational needs and good school attendance

*RFK* residual kidney function, *LVH* left ventricular hypertrophy, *PTH* parathyroid hormone, *BP* blood pressure, *SDS* standard deviation score, *PD* peritonal dialysis, *UF* ultrafiltration

#### Assessment of access adequacy

Most paediatric nephrologists would like to participate in the fistula first policy, but it is necessary to have a culture of acceptance of AV fistula in staff and patients and trained surgical staff within an HD unit for this to be successful. Play therapy is needed to enable needling in the younger child. Assessment of the percentage of patients dialysed with an AV fistula is therefore an important way of auditing access provision. Infection rates and access failure can be assessed for both AV fistulae and CVLs.

### Preservation of residual kidney function (RKF)

Another test of optimum dialysis is whether everything possible is being done to preserve RKF. Residual kidney function clears uraemic toxins that are not removed by conventional dialysis, including renal tubular secretion of protein-bound molecules. Overall, patients with RKF have better volume control, better phosphate and potassium clearance, lower requirements for erythropoietin and better quality of life [6]. In adults, even 1 ml/min/1.73m^2^ renal urea clearance is more beneficial than increasing Kt/V by dialysis [7] and is associated with approximately a 50% decrease in mortality [6].

There are two ways that we may be able to protect RKF. The first is the avoidance of nephrotoxic medications and the second is avoidance of intradialytic hypotension, principally during HD, but also during PD [19] Large drops in BP decrease renal blood flow and may result in death of renal tissue where blood flow is already precarious. It has been suggested that fluid removal during HD should be < 13 ml/kg/h in order to prevent intradialytic hypotension [1, 14] so it would seem sensible to use this figure for avoidance of adverse effects on the kidney as well, although there is no data to substantiate this figure. As dialysis products have become more biocompatible and water quality has improved, inflammation resulting from these causes is no longer a significant cause of loss of RKF [6].

The use of diuretics to increase urine output is associated with improved sodium and potassium excretion as well as fluid control [6]. Even 50 ml extra urine a day, i.e. 350 ml per week, can be useful when urine output is very low [19]

#### Assessment of preservation of RKF

Regular and sequential urine output measurements in children are fraught with difficulties, but are the best estimate of RKF if possible. Weighing of nappies is one way this can be done. Assessment of UF rates, ensuring that they do not exceed the recommended 13 ml/kg/h, may help in the preservation of RKF. Bioimpedance spectroscopy and lung US may help in determining target weight and blood volume monitoring thereby avoiding intradialytic hypotension [20]The percentage of patients that are on, or who tried, diuretics can ensure that this aspect of increasing urine output has been assessed.

### Maintenance of target weight with normal BP, no anti-hypertensive medications and no LVH

Removal of all the fluid that has been ingested between dialysis sessions can be a difficult problem, particularly if there has been deviation from the recommended intake allowance. Chronic fluid overload causes hypertension and LVH, which results in decreased capillary density, coronary reserve and subendocardial perfusion, arrhythmia, myocardial fibrosis and eventually myocyte death and systolic and diastolic dysfunction. These changes are the cause of the very high mortality due to CVD that is seen in patients on dialysis [1, 21].

**Fig. 2:**
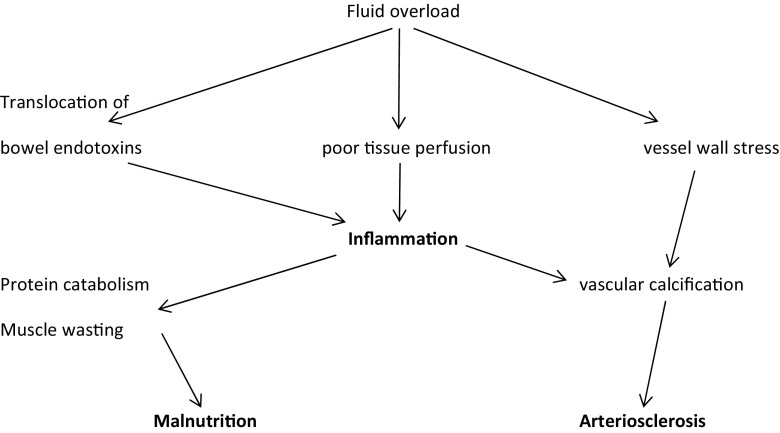
The malnutrition, inflammation, arteriosclerosis syndrome (MIA complex)

Interdialytic fluid accumulation is often on a background of an already raised extra cellular fluid (ECF). The inability to remove all accumulated fluid during dialysis contributes to inflammation and extracapillary leak, which is worsened by the frequent presence of hypoalbuminemia. Elevated ECF at the end of dialysis is associated with inflammation which promotes protein catabolism and muscle breakdown, possibly as a result of immune activation resulting from poor tissue perfusion and bowel oedema-induced translocation of bowel endotoxins into the circulation. Inflammation itself then increases vessel permeability, creating a vicious circle. Uraemic inflammation is associated with vascular calcification and atherosclerosis. These interlinking factors have been called the malnutrition, inflammation, arteriosclerosis syndrome (MIA complex) (Fig. 2) [22].

The best way to manage fluid overload is to prevent its development in the first place. Once present, particularly if chronic, it is very difficult to resolve with conventional dialysis. If ultrafiltration volumes on HD are removed at a rate in excess of 13mls/kg/h, myocardial damage can result [1, 14]. This is because areas of myocardium become hypoxic and as a result do not contract normally (left ventricular wall motion abnormalities). In the initial stages, there is usually recovery, and this has been termed ‘myocardial stunning’, but over time, the areas develop permanent myocardial dysfunction, known as ‘myocardial hibernation’ [23]. This process contributes to the loss of RKF as well. Larger UF volumes are associated with longer recovery times post dialysis [1]. Both symptoms and myocardial/renal injury may be alleviated by isolated UF. Removal of fluid by PD is limited but can be enhanced by the use of dialysate containing glucose polymers (e.g. icodextrin) as a daytime dwell.

#### Assessment of volume status and its management

Patients need access to a dietician to educate them in restricting their salt intake. Target weight should be regularly reassessed, particularly in the rapidly growing very young child. BP SDS should be aimed at within the normal range at the start of a dialysis session. ABPM is more informative than pre- and postdialysis BPs and improves the predictability of BP as a risk factor for target organ damage [24]. Anti-hypertensive medications are rarely required with effective salt and water management. The percentage of patients who have LVH on ECHO is a good assessment of successful fluid management and body fluid status.

### No discomfort during dialysis, including pain on filling with PD or intradialytic hypotension

Abdominal pain during PD can be severe and is seen much less commonly when physiological pH solutions are used. Pain on filling can also be helped by a tidal PD regimen. Symptoms on HD are most commonly related to high UF rates required for large intradialytic weight gains, leading to headaches, cramps and slow recovery after the session. The Crit-line measures real-time haematocrit and oxygen saturation during a haemodialysis session and calculates the changes in blood volume. This enables intervention during dialysis to maximise fluid removal in the minimum time without complications. When combined with bioimpedance analysis, the clinical assessment of fluid status is enhanced [20, 25].

#### Assessment of complications of the dialysis process

Percentage of patients with abdominal pain on PD and the incidence of symptoms due to intradialytic hypotension on HD in association with UF rates > 13 ml/kg/h

### Haematological and biochemical disturbances

Good dialytic clearance and attention to dietary intake and medications should enable good biochemical control. Perhaps the most important of all molecules is plasma phosphate, and the maintenance of normal plasma phosphate is one of the most important ways we have to protect the vasculature. Plasma phosphate is strongly linked with mortality from CVD in adults and with surrogate markers of CVD, such as carotid intima-media thickness (CIMT) and pulse wave velocity (PWV) in children [26]. It is also directly responsible for stimulation of PTH and CKD-MBD [27]. Dietary phosphate restriction and phosphate binders are required in virtually all children on dialysis except for those on daily treatments as phosphate clearance depends principally on dialysis time [21]. Phosphate removal can be improved on automated PD by using a daytime fill in addition to the overnight programme. Hyperparathyroidism (along with acidosis and anaemia) is also associated with poor growth [28].

The accumulation of middle molecules results in toxicity. The best known middle molecule is beta 2 microglobulin, a cause of dialysis amyloid. Despite improved clearance with high flux dialysers and HDF, reports of benefits to prevention of amyloid have been variable, and it is not clear whether there is value in routine measurement of this molecule [1]. Protein-bound molecules are particularly difficult to remove by dialysis. Phenols and indoles, which originate from the intestinal microbial metabolism of dietary amino acids, have been used to represent removal of these large molecule toxic metabolites. More frequent dialysis or longer dialysis schedules have not shown any consistent benefits, so these molecules are not routinely measured at present but may become important tools in the future [6].

#### Assessment of haematological and biochemical management

These factors can be easily audited alongside the availability of dietary advice.

### Good nutrition and growth

Fifty percent of children have a height below the normal range when starting RRT and in the majority HtSDS on dialysis declines thereafter. There is a huge variation in Ht SDS around the world as demonstrated by the International Paediatric Dialysis Network (IPDN) [14]. Therapies that have been shown to be beneficial to growth include attention to nutrition with regular consultation by a dietician, and in particular provision of gastrostomy feeds in infants, the use of physiological PD fluids and growth hormone [29].

#### Assessment of nutrition and growth

Maintenance of a normal albumin and a urea below 20 mmol/l demonstrates good dietetic control. The percentage of patients seeing a dietician, mean and range Ht SDS and Wt SDS for all patients and their tracking for individuals, early use of enteral feed supplements when intake is inadequate and/or the growth rate is declining and the percentage of such patients with gastrostomies are objective measures of good nutritional management. The KDOQI review of nutritional management of children with CKD gives guidelines on the frequency and types of assessments needed at different ages [30].

### Hospitalisation rates

The US Renal Data System (USRDS) for 2008–2012 reported that the one-year hospitalisation rate in all children on RRT was 2005 admissions per 1000 patient years. Rates vary with age: they were highest in the youngest children, aged 1–4 years, at 3253 admissions per 1000 patient years, then decreased progressively, rising again to 1988 admissions per 1000 patient years in the 18–21 year age group. The principal cause for admission was infection, occurring in 606 per 1000 patient years. Cardiovascular (CV) events was the second commonest cause, with an admission rate of 374 per 1000 patient years. Children on HD had the highest rates of admission for CV events, and those on PD, the highest rates for infection [31].

A recent study from Turkey has shown a hospitalisation rate of 1.7 episodes per person per year. The first and second commonest causes were peritonitis and then volume overload [10]. Anaemia has been shown to be associated with hospitalisation both in this study, and in registry data from the USA in 1659 children on dialysis: the adjusted relative rate of all-cause hospitalisations was significantly lower in children with a haemoglobin maintained above 12 g/dl (0.81, 0.74–0.89) [32].

Readmission within 30 days of discharge has been used as a measure of quality of care. Approximately one quarter to one third of patients with ESKD age 0–19 years are readmitted to the hospital within 30 days of discharge. Readmission, like admission, is more common in young children, and also in those receiving HD. Approximately 50% of the readmissions were for a similar diagnosis as the index admission, suggesting the possibility of inadequate treatment of the index episode [33].

#### Assessment

Hospitalisation rates and their causes are easily obtained when computerised systems are available.

### Feels well, attends school

HRQoL scores are lower in children on dialysis than in most other chronic conditions except cancer and depression is prevalent. Parental stress due to responsibilities of provision of care, concerns about their potential role in complications experienced by their child and the trauma associated with watching their child undergo invasive procedures is a common issue. Frequent absenteeism from school for dialysis needs or hospitalisation is reported [14]. Many children on dialysis have special educational needs, such as hearing and visual difficulties. There is a high incidence of cognitive difficulties, with executive function and attention being the principal areas of concern [34].

#### Assessment of well-being and school attendance

There are well recognised, standardised assessments of health-related quality of life (HRQoL), and indeed in the USA, all dialysis patients are mandated to have a HRQoL assessment performed annually. Such assessments include the child and carer [34]. A psychologist-directed assessment is a way to target educational difficulties and develop strategies to overcome them. Dialysis sessions outside school hours should be tailored whenever possible. Liaison between the unit teaching staff and the school enables an integrated approach to the child’s education. School attendance is monitored and easily obtainable. Clearly therefore, all children need access to social workers, psychologists and play therapists as required.

## Conclusion

Many different factors are important in the provision of optimum dialysis. In order to successfully deliver this complex therapy, most important of all is that there is a fully trained and committed multidisciplinary team of paediatric nephrologists, renal nurses, renal dieticians, transplant surgeons, urologists, interventional radiologists, anaesthetists, pharmacists, play specialists, schools teachers, psychologists and social workers, all of whom play an essential role in the provision of the best possible care to our patients.

## Multiple-choice questions (answers are provided following the reference list)

1. Kt/V_urea_
  - Is not affected by patient size
  - Is an assessment of middle molecule clearance
  - Can be used to assess small solute clearance
  - Correlates with residual kidney function
  - Is consistently correlated with patient outcome
2. Dialysis should be started when:
  - The urea is over 55 mmol/l, but other biochemistry and urine output are normal
  - The GFR is 5 ml/min/1.73m^2^ but the child is asymptomatic and urine output is maintained
  - The GFR is 11 ml/min/1.73m^2^ and retransplantation will not be possible
  - The GFR is 8 ml/min/1.73m^2^ and there is resistant anaemia
  - The GFR is 10 ml/min/1.73m^2^ but there is decreasing urine output and salt and water retention causing difficult to treat hypertension
3. Dialysis access
  - Insertion of a Tenckhoff catheter is a straight forward surgical procedure that can be undertaken by a surgeon without specific training
  - Leakage of PD fluid along the catheter track does not usually cause infection and resolves spontaneously
  - Children aged 3 years are too young for an AV fistula, even in the hands of experienced staff
  - Central venous line infection can lead to vessel stenosis
  - Children with AV fistulae are admitted to hospital more than those with a central venous line
4. Residual kidney function
  - Allows renal tubular secretion of protein bound molecules
  - Does not influence volume control, phosphate or potassium clearance
  - Does not benefit mortality
  - Is classically reduced by episodes of hypertension
  - Diuretics will not help urine output
5. Intradialytic fluid accumulation
  - Is often on a background of an already raised ECF
  - Is driven by the patient’s primary drive for water rather than salt ingestion
  - Elevated ECF at the end of dialysis is associated with inflammation which promotes protein anabolism
  - Is best removed by rapid UF during dialysis
  - Does not contribute to hypertension
